# Synthesis and Characterization of the *in Situ* Bulk Polymerization of PMMA Containing Graphene Sheets Using Microwave Irradiation

**DOI:** 10.3390/molecules18033152

**Published:** 2013-03-11

**Authors:** Mohammad A. Aldosari, Ali A. Othman, Edreese H. Alsharaeh

**Affiliations:** 1 King Abdulaziz City for Science & Technology (KACST), National Nanotechnology Research Center (NNRC), P.O. Box 6086, Riyadh 11442, Saudi Arabia; E-Mail: aldosari@kacst.edu.sa; 2 College of Science and General Studies, Alfaisal University, P.O. Box 50927, Riyadh 11533, Saudi Arabia; E-Mail: aothman@alfaisal.edu

**Keywords:** graphene, graphene oxide, polymer, methyl methacrylate, microwave synthesis

## Abstract

Polymethylmethacrylate–graphene (PMMA/RGO) nanocomposites were prepared via *in situ* bulk polymerization using two different preparation techniques. In the first approach, a mixture of graphite oxide (GO) and methylmethacrylate monomers (MMA) were polymerized using a bulk polymerization method with a free radical initiator. After the addition of the reducing agent hydrazine hydrate (HH), the product was reduced via microwave irradiation (MWI) to obtain R-(GO-PMMA) composites. In the second approach, a mixture of graphite sheets (RGO) and MMA monomers were polymerized using a bulk polymerization method with a free radical initiator to obtain RGO-(PMMA) composites. The composites were characterized by FTIR, ^1^H-NMR and Raman spectroscopy and XRD, SEM, TEM, TGA and DSC. The results indicate that the composite obtained using the first approach, which involved MWI, had a better morphology and dispersion with enhanced thermal stability compared with the composites prepared without MWI.

## 1. Introduction

Graphene (GR) is the thinnest two-dimensional graphitic carbon (sp^2^-bonded carbon sheet) material known, being one atom in thickness [1,2,3]. GR has recently attracted much interest as a filler for the development of new nanocomposites [4,5,6,7,8,9,10,11,12,13,14]. Its extraordinary structural, mechanical, thermal, optical and electrical properties make GR an excellent two-dimensional filler material for polymer composites for applications in many technological fields [4,5,6,7,8]. 

One of the greatest challenges for this is achieving good dispersion of the nanoscale filler in the composites. Good dispersion is crucial for achieving the desired enhancement in the final physical and chemical properties of the composites [2], especially for GR, which has a strong tendency to agglomerate due to intrinsic van der Waals forces [15]. Various techniques have been developed for the synthesis of these nanocomposite structures, including solution mixing, melt blending, *in-situ* microwave irradiation (MWI) and *in-situ* polymerization [16,17,18,19,20,21]. 

One of the advantages of graphene oxide (GO) is that it can be easily dispersed in water and physiological environments due to its abundant hydrophilic groups, which include hydroxyl, epoxide and carboxylic groups on its large surface [22,23]. Recently, the El-Shall group demonstrated a novel approach for the production of GR sheets (RGO) prepared via the Hoffman Hummers method via reduction of GO using hydrazine hydrate (HH) facilated by using MWI. The resulting product is composed of graphene sheets with polar functional groups, even after the reduction [24]. GO has an affinity for polar solvents and polymers [25]. This affinity makes GO an important intermediate in the preparation of RGO polymer composites via chemical reduction [21]. In this approach, oxygen functional groups, such as carboxyl, carbonyl and hydroxyl groups, were introduced into the carbon skeleton of the GO [25,26], enabling it to interact with polymers through those oxygenated functionalities [27,28,29,30,31]. This interaction has been demonstrated during the preparation of polymer nanocomposites, in which the dispersion of chemically reduced GO is maintained in the presence of the polymer [23,24,25,26,27,28,29]. We are interested in investigating the use as a filler of RGO nanosheets prepared via the reduction of GO facilated by MWI, which offers a fast and easy method for synthesizing polymer nanocomposites [16]. In MWI, dielectric heating energy is transferred directly to the reactants, and the energy is supplied to the molecules faster than they are able to relax, creating high instantaneous temperatures that typically increase the yield and quality of the product [13].

Polymethylmethacrylate (PMMA) is an important polymeric material that has been used in medicine (bone cement), dentistry, the paper, paint and automobile industries and in many other applications [22,23,24,25,26,32]. Several studies have reported the successful incorporation of GR nanosheets into the PMMA matrix with different preparation techniques using various methods of GR preparation, such as from reduced GO, functionalized RGO sheets or chemical vapor deposition [17,18,19,33]. Herein, we report two different preparation techniques for polymethylmethacrylate (PMMA/RGO) nanocomposites. The first technique involves preparing the RGO nanosheets filler via the MWI reduction of GO in the presence of HH. The second technique involves *in situ* reduction of GO/PMMA via MWI in the presence of HH. Our goal is to understand the effect of nanocomposite preparation techniques on the homogeneity and thermal behavior of RGO-polymer nanocomposites. The general approach focused on a facile method for *in situ* bulk polymerization of polymethylmethacrylate containing RGO nanosheets (RGO/PMMA). In the first approach, a mixture of GO and methylmethacrylate monomers (MMA) were polymerized using a bulk polymerization method. After the addition of a reducing agent, HH, the product was reduced using conventional microwave irradiation. In the second approach, a mixture of RGO, which was produced via MWI and MMA monomers were polymerized using a bulk polymerization method using a free radical initiator to obtain RGO-(PMMA) composites. The composites were characterized by FTIR, ^1^H-NMR and Raman spectroscopy and XRD, SEM, TEM, TGA and DSC. The results indicate that the composite obtained using the first approach, which involved MWI, had a better morphology and dispersion with enhanced thermal stability compared with the composites prepared without MWI. 

## 2. Results and Discussion

**Figure 1:**
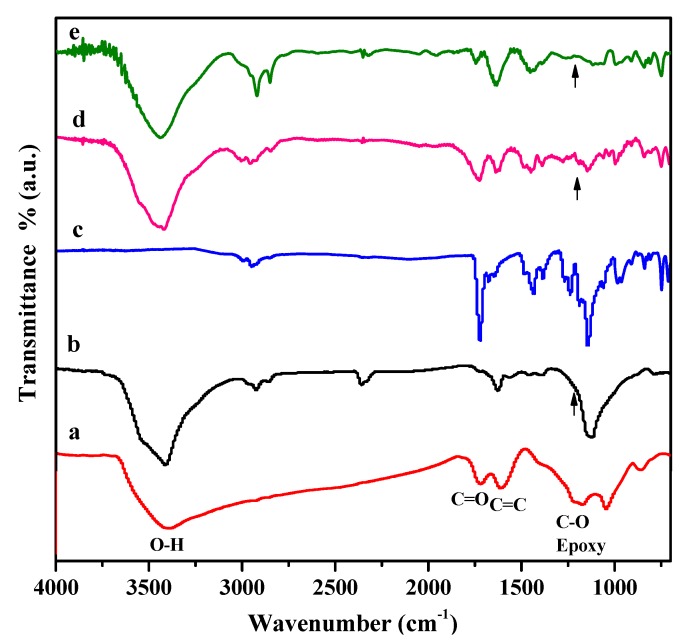
FTIR spectra of (**a**) GO; (**b**) RGO; (**c**) neat PMMA; (**d**) RGO-(PMMA) nanocomposites and (**e**) R-(GO-PMMA) nanocomposites.

FTIR spectral analysis was performed to confirm the chemical structure of all of the RGO/poly(methyl methacrylate) (PMMA) nanocomposites. Figure 1 summarizes the FTIR spectra of the GO, RGO, neat PMMA, RGO-(PMMA) and R-(GO-PMMA) nanocomposites. The characteristic FTIR features of GO (Figure 1a) include the presence of different types of oxygen functionalities, which have been confirmed by the band at 3420 cm^−1^, which corresponds to the O-H group, the bands at 1720 and 1618 cm^−1^, which correspond to the C=O carbonyl/carboxyl and C=C aromatic groups, respectively, and the band at 1220 cm^−1^, which corresponds with the C-O in the epoxide group [30,34]. For the RGO, Figure 1b indicates that the O-H band at 3430 cm^−1^ was reduced in intensity due to the deoxygenation of the GO-oxygenated functionalities. The spectrum of RGO also contains bands at 1627 cm^−1^ and 1139 cm^−1^, which correspond to C=C and C-O groups, respectively. For the neat PMMA (Figure 1c), the spectrum showed characteristic bands at 2965, 2847 and 1730 cm^−1^, which correspond to the aliphatic C-H, -CH_2_ and C=O groups, respectively. The spectrum also had a band at 1150 cm^−1^ that has been assigned to the C-O-C group. The bands between 1270 cm^−1^ and 990 cm^−1^ originate from the C-O group. For the RGO-(PMMA) nanocomposites, Figure 1d shows the characteristic bands at 3420, 1726 and 1620 cm^−1^ that correspond to the O–H, C=O and C=C groups, respectively [13]. When MWI was employed in the preparation of R-(GO-PMMA) nanocomposites (Figure 1e), there was an increase in the intensity of the C=C bands and a decrease in the intensity of the C=O bands. In addition, strong characteristic bands associated with aliphatic C-H and -CH_2_ groups were observed at 2921 and 2854 cm^−1^, respectively, in the spectrum of the R-(GO-PMMA) nanocomposites compared with that obtained by the *in situ* method (Figure 1d), which indicates the presence of polymer chains despite the use of HH. In summary, the FTIR results suggest that all of the nanocomposites exhibit the characteristic peaks for PMMA chains and RGO sheets. 

**Figure 2:**
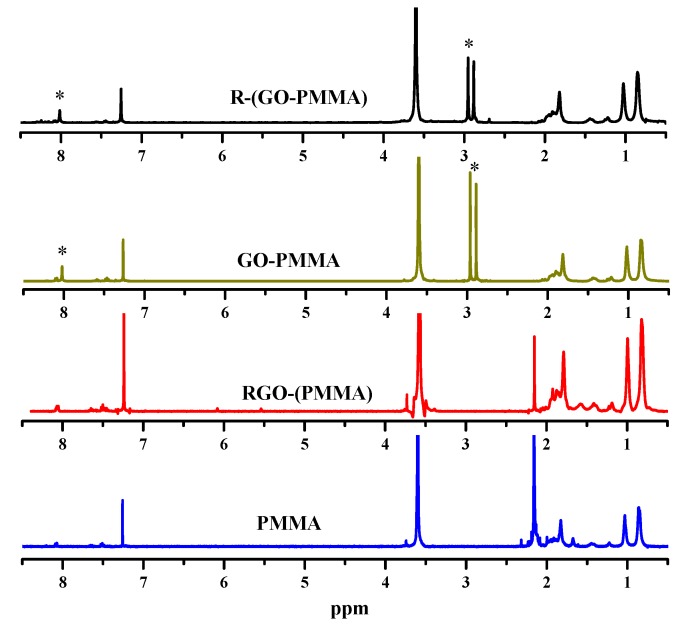
^1^H-NMR spectra of neat PMMA and all of the nanocomposites prepared from *in situ* bulk polymerization and MWI [* indicates residual solvent (DMF) peaks].

Additional structural evidence to verify the success of polymerization and preparation of the RGO/poly(methyl methacrylate) (PMMA) nanocomposites can be obtained from ^1^H-NMR data. The ^1^H-NMR spectra of the neat PMMA, RGO-(PMMA), GO-PMMA and R-(GO-PMMA) nanocomposites are shown in Figure 2. The peaks at δ = ~0.6–1.0 ppm correspond to -CH_3_, and those between δ = ~1.3 and 2.0 ppm correspond to -CH_2_. It has been reported that PMMA exhibits a band at δ = 2.8–3.6 ppm that corresponds to the methoxy ester linkage (–COOCH_3_) [13,35] these peaks were observed for the polymer and its RGO nanocomposites using the different preparation methods. 

**Figure 3:**
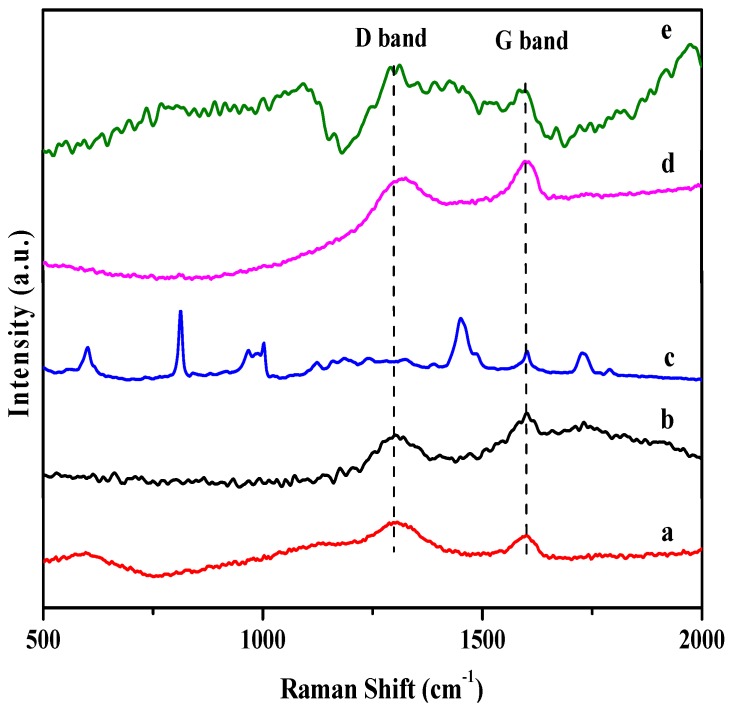
Raman spectra of (**a**) GO; (**b**) RGO; (**c**) neat PMMA; (**d**) RGO-(PMMA) nanocomposites and (**e**) R-(GO-PMMA) nanocomposites.

Raman spectroscopy was also employed to characterize the RGO/PMMA nanocomposites. Raman spectroscopy is a very powerful tool that provides essential information for evaluating the covalent modification of RGO nanosheets [19]. Figure 3 displays the Raman spectra of GO, RGO, neat PMMA and PMMA prepared *in situ* bulk polymerization and MWI. A comparison of the intensity of the characteristic D band at 1300 cm^−1^ and the G band at 1600 cm^−1^ is a well-accepted method for estimating the quality and structural order of graphitic structures [7,8,13]. The D band is related to the sp^3^ states of carbon and is used as proof of disruption in the aromatic π-electron system in RGO. In addition, the G band at 1600 cm^−1^ is related to the sp^2^ state of carbon [19]. The D/G ratio in the GO, RGO, RGO-(PMMA) and R-(GO-PMMA) nanocomposites is summarized in Table 1. The ratio of the intensities of the two bands (D/G) should increase as a result of the interaction between the π network in RGO and the ester linkage group -COOCH_3_ in PMMA [9,13]. The results clearly showed an increase in the intensities of the (D/G) band ratios of RGO (0.66), RGO-(PMMA) (0.93) and R-(GO-PMMA) (1.31). This result indicates that the sp^2^-hybridized carbons were converted to sp^3^ hybridized carbons, which may be due to the covalent attachment of the RGO sheets to the PMMA [36]. The ratio was the highest for the R-(GO-PMMA) nanocomposites, indicating a stronger covalent interaction than in the RGO-(PMMA) nanocomposite, which was prepared via *in situ* bulk polymerization. 

**Table 1:** Summary of the D/G ratio determined from the Raman spectroscopy.

Sample	D band 1299 cm^−1^	G band 1600 cm^−1^	D/G ratio
GO	0.052	0.036	1.44
RGO	0.016	0.025	0.64
PMMA	---	---	---
RGO-(PMMA)	0.988	1.066	0.93
R-(GO-PMMA)	0.021	0.016	1.31

**Figure 4:**
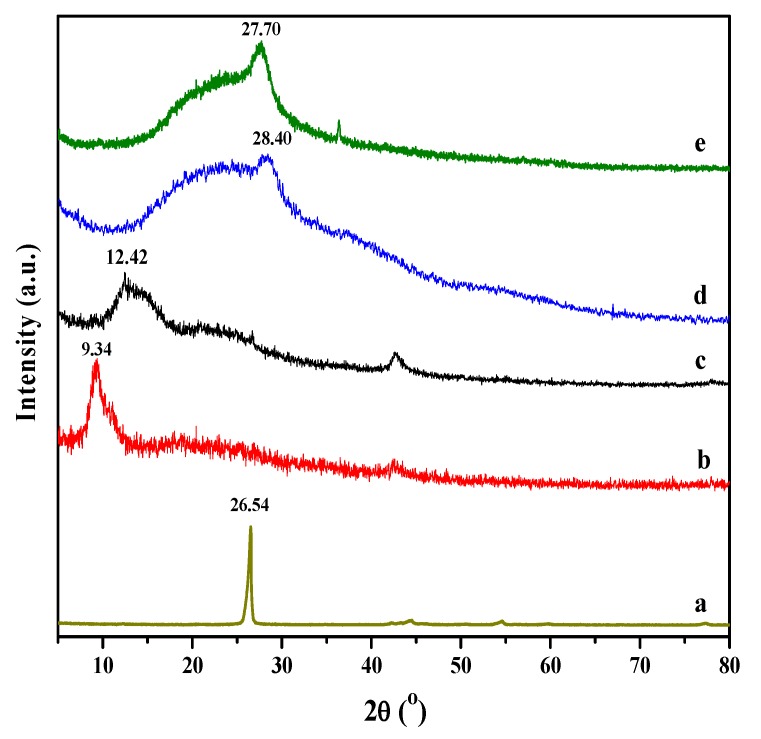
XRD patterns of (**a**) Graphite; (**b**) GO; (**c**) RGO; (**d**) RGO-(PMMA) nanocomposites and (**e**) R-(GO-PMMA) nanocomposites.

The presence, intercalation and/or exfoliation of RGO sheets in the PMMA matrix can be evaluated using XRD. Figure 4 displays the XRD patterns for graphite, GO, RGO, neat PMMA, RGO-(PMMA) and R-(GO-PMMA). The XRD pattern of the graphite displayed in Figure 4a showed a strong characteristic peak at 2θ = 26.54°, with a d-spacing of 0.34 nm. The XRD pattern of GO displayed in Figure 4b showed a characteristic peak (2θ) at approximately 9.34°, which corresponds with a d-spacing of 0.95 nm. After GO was reduced by HH (Figure 4c), the d-spacing decreased. In addition, the peak appeared at 2θ = 12.42°, with a d-spacing of 0.71 nm. This result confirms the chemical reduction of GO and formation of RGO via the HH reducing agent [37,38]. In addition, this result also indicates the removal of large number of oxygen-containing groups and the formation of much more exfoliated RGO sheets, as well as a change in the hybridization of the reduced carbon atoms from tetrahedral sp^3^ to planar sp^2^ [39,40]. The XRD pattern of the RGO-(PMMA) nanocomposites (Figure 4d) had a broad peak, indicating an amorphous structure, which corresponds primarily with the PMMA with a 2θ of 28.40° and a d-spacing of 0.32 nm. The XRD pattern of the prepared R-(GO-PMMA) nanocomposites prepared via MWI (Figure 4e) showed an increase in the d-spacing, with a band at 2θ = 27.70° and a d-spacing of 0.33 nm. The shift in the d-spacing in the R-(GO-PMMA) nanocomposites indicates that the interlayer spacing increased due to various degrees of intercalation, which suggest that various oxygen-containing functional groups (*i.e.*, carboxyl, epoxy and hydroxyl) on the RGO sheet planes were introduced in a larger quantity by MWI. These oxygen-containing functional groups enhanced the interaction along the edges of the nanosheets resulting in an enlargement in the interlayer spacing of RGO/PMMA. The broad peaks near 19° in the R-(GO-PMMA) spectrum showed less broadening compared with the RGO-(PMMA) nanocomposite, indicating agglomeration in these nanocomposites. This agglomeration may arise from strong van der Waals interactions between the reduced GO sheets [41]. In addition, the characteristic peaks of RGO and GO do not appear in the patterns of the nanocomposites, which indicates that the RGO layers were exfoliated in the composites [39,42,43].

**Figure 5:**
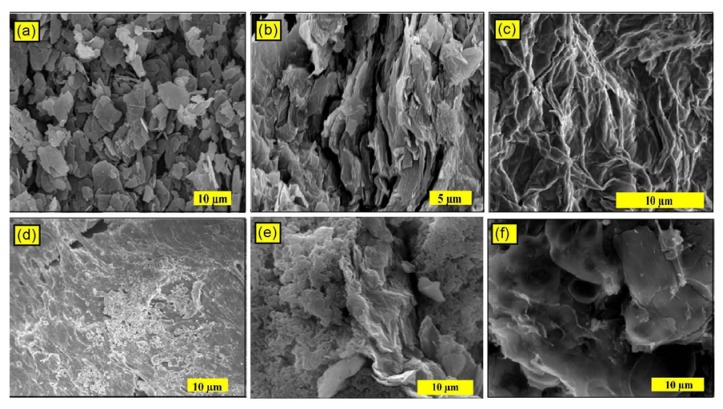
The SEM micrographs of (**a**) graphite; (**b**) GO; (**c**) RGO; (**d**) neat PMMA; (**e**) RGO-(PMMA) nanocomposites; and (**f**) R-(GO-PMMA) nanocomposites after reduction by MWI.

Direct evidence for the exfoliation of the RGO in the final polymer nanocomposites can be obtained from SEM and TEM, which can also provide images of the dispersion in the RGO layers in the PMMA matrix. Figure 5a shows the SEM image of the graphite. The particles have a plate-like shape with average sizes of 1–10 µm. The prepared GO (Figure 5b) was not fully exfoliated and had a flaky texture. This result suggests a partially exfoliated structure and reflects its layered microstructure containing large interlayer spacing and thick multilayer stacks, which is in agreement with the literature [44]. Figure 5c shows the SEM image of RGO, which reveals that the RGO consisted of randomly aggregated thin crumpled sheets that are closely associated with each other, forming a disordered solid [45]. The SEM image of the RGO-(PMMA) nanocomposites prepared via the *in situ* method (Figure 5e) shows that RGO is stacked up, and not well dispersed in the PMMA matrix. For the R-(GO-PMMA) nanocomposites (Figure 5f), the wrinkled and crumpled profile of RGO was observed with significant changes in the morphology compared with neat PMMA (Figure 5d) and RGO-(PMMA) (Figure 5e). 

**Figure 6:**
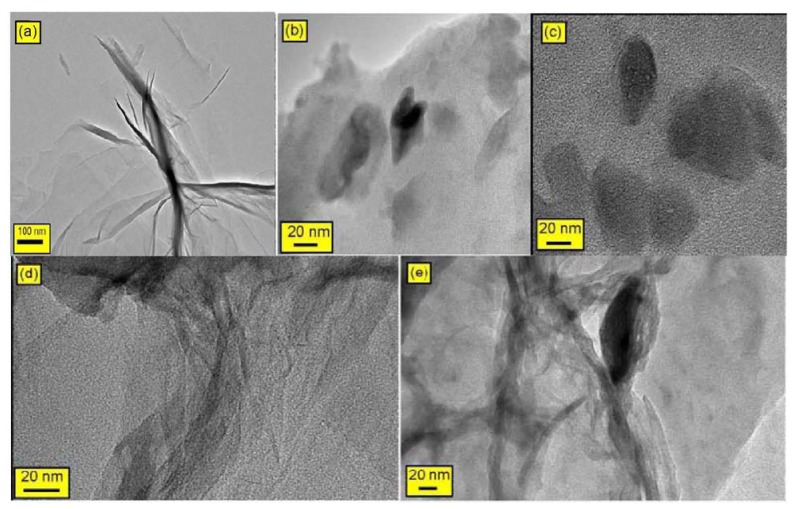
The TEM micrographs of (**a**) RGO; (**b**) neat PMMA; (**c**) GO-PMMA; (**d**) RGO-(PMMA) nanocomposites; and (**e**) R-(GO-PMMA) nanocomposites.

Because SEM cannot spatially resolve the thickness of an individual RGO-based sheet, transmission electron microscopy (TEM) was employed to determine if the RGO-based sheets were indeed present in the composites as single exfoliated sheets or as multi-layered sheets [6]. TEM offers direct evidence for the formation of the RGO nanosheets on the PMMA nanocomposites. Figure 6 displays the TEM images of all of the composites and confirm the presence of RGO sheets in the PMMA matrix. In the case of R-(GO-PMMA), Figure 6e, it is clearly shown that RGO is better dispersed into the PMMA matrix compared to the case of RGO-(PMMA) (Figure 6d). The XRD, SEM and TEM results suggest that the RGO sheets were dispersed in the PMMA matrix, which indicates good compatibility between the nanosheet and polymer matrix. This result may be attributed to the covalent interactions between the RGO nanosheet and PMMA matrix [18,19,46,47]. The de-lamination, intercalation and exfoliation of the RGO sheets was enhanced and improved with MWI and showed better dispersion of the RGO filler in the PMMA matrix, which should enhance the thermal properties of the RGO-(PMMA) nanocomposites.

**Figure 7:**
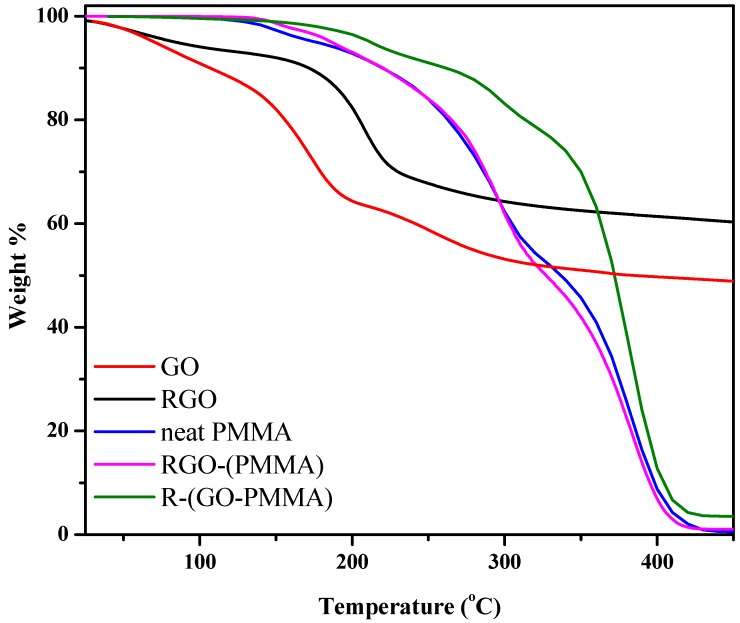
TGA thermograms of neat PMMA, RGO-(PMMA) and R-(GO-PMMA) nanocomposites.

To investigate the effect of the extent of interaction between the PMMA matrix and RGO on the composite properties, we compared the thermal degradation of the polymer itself with the composites. The thermal degradation studies of the GO, RGO, neat PMMA, RGO-(PMMA) and R-(GO-PMMA) nanocomposites were performed using thermogravimetric analysis (TGA) under a N_2_ atmosphere. The results are displayed in Figure 7 and summarized in Table 2. The results indicated that GO (Figure 7a) was not thermally stable and exhibited a significant mass loss (22%) at 160 °C due to the pyrolysis of the oxygen-containing functional groups generating CO, CO_2_ and steam [48,49]. However, the TGA curve of the RGO (Figure 7b) showed enhanced thermal stability compared with GO due to the removal of oxygen-containing functional groups by hydrazine reduction.

The TGA results of the RGO-(PMMA) nanocomposites showed a decrease of 9 °C in the maximum degradation temperature (temperature at 50% mass loss, *T*_max_) from 335 to 326 °C compared with the neat PMMA. For the R-(GO-PMMA) nanocomposites (Figure 7e), the curve indicated that they were more thermally stable, with a maximum degradation temperature (*T*_max_ = 372 °C) that was 37 °C higher than that of neat PMMA. We attributed this result to the homogenous dispersion of the RGO filler within the PMMA matrix, and this homogeneity was enhanced by MWI. 

To further understand the thermal behavior and homogeneity of the nanocomposites prepared by the two different methods, differential scanning calorimetry (DSC) of the neat PMMA, RGO-(PMMA) and R-(GO-PMMA) nanocomposites was employed to compare the glass transition temperature (*T*_g_) of the polymer itself and with the nanocomposites. 

**Table 2:** Summary of the thermal behavior data obtained from TGA and DSC measurements.

Sample	*T_d_* ^a^ (°C)	*T_max_* ^b^ (°C)	*T_g_* (°C)
GO	107	160	---
RGO	172	210	---
PMMA	290	335	117
RGO-(PMMA)	295	326	94
R-(GO-PMMA)	299	372	120

**a:** The degradation temperature at a 10% weight loss in the decomposition stage. **b:** The maximum degradation temperature at a 50% weight loss in the decomposition stage.

**Figure 8:**
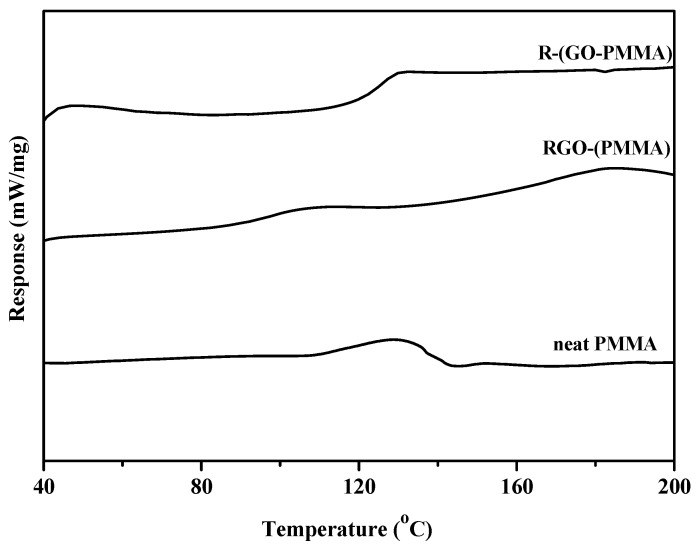
DSC thermograms of neat PMMA, RGO-(PMMA) and reduced R-(GO-PMMA) nanocomposites.

The *T*_g_ values obtained from the DSC thermograms are displayed Figure 8 and summarized in Table 2. For the RGO-(PMMA) nanocomposites (Figure 8b), the results showed that the *T*_g_ value of the nanocomposites after loading the RGO decreased significantly (by 23 °C). This value is lower than that of the neat PMMA (Figure 8a). However, when we used the second approach in which the nanocomposites were prepared using MWI, the R-(GO-PMMA) nanocomposites (Figure 8c) showed a significantly improved thermal stability, with a *T*_g_ of 120 °C, which 26 °C higher than that of the RGO-(PMMA) nanocomposites and 3 °C than that of the neat PMMA. This result suggests a strong interaction between the PMMA chains and RGO. 

Previous work has shown that the interfacial strength between nanofillers and polymers and consequently, the thermal properties of nanocomposites, can be altered by varying the sample preparation method [37,50]. In this work, when MWI was used, the *T*_g_ increased upon addition of the RGO nanofiller, which may be due to either restriction in the chain mobility resulting from the confinement effect of the 2D-layered RGO incorporated into the matrix or strong nanofiller-polymer interaction [51,52,53]. Therefore, good dispersion without agglomeration of RGO may result from the fast thermal reduction process that is offered by MWI. When we employed the *in situ* bulk polymerization approach to prepare the RGO-(PMMA) nanocomposites, the *T*_g_ decreased, which may be associated with the agglomeration of the RGO nanosheets due to the van der Waals forces between the RGO layers, leading to an increase in the mobility of the polymer chain resulting from an increase in the free volume, which has been observed with the addition of a plasticizer [50,53,54].

## 3. Experimental

### 3.1. Materials

Extra pure graphite powder (>99.5%) was purchased from Merck (Darmstadt, Germany), and hydrazine hydrate (HH, 80%) was obtained from Loba Chemi. Pvt. Ltd (Mumbai, India). Methyl methacrylate (MMA, Acros Chemical Co., Loughborough, UK, 99%) was kept in a refrigerator and used as received. Benzoyl peroxide (BP, BDH Chemicals Ltd., East Yorkshire, UK) was used as an initiator. Potassium permanganate (KMNO_4_, >99%) and hydrogen peroxide (H_2_O_2_, 30%) were obtained from Merck. Other solvents and chemicals were of analytical grade and used without further purification.

### 3.2. Preparation of Graphite Oxide (GO)

GO was synthesized by the oxidation of graphite powder via the Hummers and Offeman method [55]. Natural graphite (3.5 g) was added to 98% H_2_SO_4_ (100 mL) under vigorous stirring. Next KMNO_4_ (10 g) was slowly added, and the temperature was maintained below 20 °C. The stirring was continued for 2 h at 35 °C. Then, the content of the flask was poured into deionized water (500 mL), and a sufficient amount of H_2_O_2_ (*ca*. 20 mL of a 30% aqueous solution) was added to destroy any excess permanganate. Upon treatment with the peroxide, the suspension turned bright yellow. GO was isolated by filtration through a sintered glass filter. The product was thoroughly washed with dilute HCl and then hot water to remove the residual sulfate ions yielding a yellow-brown filter cake. After repeated washing of the resulting yellowish-brown cake with hot water, the GO was dried at 80 °C.

### 3.3. Preparation of RGO

The dried GO (400 mg) was stirred and sonicated in deionized water (20 mL) until a homogeneous yellow dispersion was obtained. The GO can be dispersed easily in water due to the presence of a variety of hydrophilic oxygen groups (OH, O and COOH) on the basal planes and edges. The solution was placed inside a conventional microwave after the addition of HH reducing agent (400 μL). The microwave oven (KenWood MW740) was operated at full power (900 W) in 30 s cycles (on for 10 s and off and stirring for 20 s) for a total reaction time of 2 min [16]. The yellow dispersion of GO gradually changed to a black color indicating the completion of the chemical reduction to RGO. The RGO sheets were separated using a centrifuge (Centurion Scientific Ltd., West Sussex, UK) operated at 5,000 rpm for 15 min and dried at 80 °C overnight.

### 3.4. Preparation of the RGO-(PMMA) Nanocomposites via the *in Situ* Method

RGO powder [2.0 (wt./wt.%)] was added to the MMA monomer, stirred and sonicated for 1 h. The benzoyl peroxide (BP) initiator (5.0 wt.%) was added to the suspension and stirred until the initiator dissolved. Then, the mixture was heated to 60 °C to initiate the polymerization using a shaking-water bath (GFL, Burgwedel, Germany). The reaction mixture was maintained at 60 °C for 20 h. After the polymerization finished, the product was poured into an excess of methanol, stirred for 15 min and washed with hot water to remove the MMA monomers. Then, the product was filtered and dried at 80 °C overnight. 

### 3.5. Preparation of the R-(GO-PMMA) Nanocomposites via the MWI Method

GO powder [2.0 (wt./wt.%)] was added to the MMA monomer, stirred and sonicated for 1 h. The benzoyl peroxide (BP) initiator (5.0 wt.%) was added to the suspension and stirred until the initiator dissolved. Then, the reaction mixture was maintained at 60 °C for 20 h to promote polymerization using a shaking-water bath (GFL). After the polymerization finished, the product was poured into an excess of methanol, stirred for 15 min and washed with hot water to remove MMA monomers. Then, the product was filtered and dried at 80 °C overnight. Four hundred milligrams of the dried composite of GO-PMMA was dissolved in DMF, stirred and sonicated for 1 h. Then, the composite was placed inside a conventional microwave oven (Kenwood MW740) following the addition of HH (400 µL). The microwave oven was operated at full power (900 W) in 30 s cycles (on for 10 s and off and stirring for 20 s) for a total reaction time of 2 min [16]. Then, the composites were separated using a centrifuge (Centurion Scientific Ltd.) operated at 5,000 rpm for 15 min and dried in an oven at 80 °C overnight. For comparison, the neat PMMA was prepared via a similar procedure in the absence of the RGO and GO. 

### 3.6. Characterization and Instrumentation

The FTIR (Thermo Scientific Nicolet-iS10, Madison, WI, USA) spectra of the composites were recorded in the range of 4000–500 cm^−1^. The ^1^H-NMR of the solutions was recorded on a Bruker Avance (III) instrument (Bruker, Milton-Ontario, Canada) at 200 MHz using CDCl_3_ as the solvent, and the composites were macerated in the solvent for 1 day. The Raman spectra of composites were measured with a Bruker Equinox 55 FT-IR spectrometer equipped with an FRA106/S FT-Raman module and a liquid N_2_-cooled Ge detector using the 1064 nm line of a Nd:YAG laser with an output laser power of 200 mW. The thermogravimetric analyses (TGA) of the composites were studied using a NETZCH 209 F1 thermogravimetric analyzer (Netzsch, Selb, Germany). The X-ray diffraction (Model PW 1729, Philips, Amsterdam, The Netherlands) of the composites were investigated with Cu radiation [30 kV, 40 mA, Kα radiation (λ = 1.54430 Å)] between 2θ of 5° and 100°. The decomposition temperature measurements using TGA were performed under an N_2_ atmosphere at a heating rate of 10 °C per minute from 25 °C to 800 °C. Differential scanning calorimetry (DSC, NETZCH 204 F1) measurements were employed to estimate the glass-transition temperature (*T*_g_) of each composite. The composites were heated from −25 °C to 100 °C at a heating rate of 10 °C per min. Then, a double run was performed after cooling at a heating rate of 2 °C per min from 25 °C to 350 °C. The *T*_g_ was taken as the midpoint of the transition. A scanning electron microscope (SEM, FEI Quanta 200, FEI, Hillsboro, OR, USA) was employed to study the morphology of the composites after they were mounted on the composite slabs and coated with gold via sputtering system (Polaron E6100, Bio-Rad, Birmingham, UK). Ultrathin sections of the composites were prepared for transmission electron microscopy (TEM) studies; the transmission electron microscope (JEOL JSM-2100F, JEOL, Tokyo, Japan) was operated at 200 kV.

## 4. Conclusions

In summary, we have successfully prepared R-(GO-PMMA) and RGO-(PMMA) using *in situ* bulk polymerization facilitated by MWI. Thermal analysis showed an enhancement in the thermal properties of the RGO/PMMA nanocomposite prepared using MWI, which indicates that the RGO sheets efficiently reinforced the PMMA matrix. Therefore, our approach is promising for the development of a new class of graphene–polymer nanocomposites.
